# Neutralization of cholera toxin by Rosaceae family plant extracts

**DOI:** 10.1186/s12906-019-2540-6

**Published:** 2019-06-20

**Authors:** Magdalena Komiazyk, Malgorzata Palczewska, Izabela Sitkiewicz, Slawomir Pikula, Patrick Groves

**Affiliations:** 1 Laboratory of Biochemistry of Lipids, Nencki Institute of Experimental Biology, 3 Pasteur Street, 02-093, Warsaw, Poland; 2 Laboratory of Molecular Interactions and NMR, Instituto de Tecnologia Química e Biológica, Av. da República, 2780-157 Oeiras, Portugal; 3 Department of Molecular Biotechnology, Chemistry Faculty, University of Gdansk, 63 Wita Stwosza Street, 80-308 Gdańsk, Poland; 4 Department of Drug Biotechnology and Bioinformatics, National Medicines Institute, 30/34 Chełmska Street, 00-725 Warsaw, Poland; 5 Department of Biomedicinal Chemistry, Chemistry Faculty, University of Gdansk, ul. 63 Wita Stwosza Street, 80-308 Gdańsk, Poland

**Keywords:** Cholera toxin, Diarrhea, Herbal remedies, Plant extracts, Rosaceae

## Abstract

**Background:**

Cholera is one of the most deadly diarrheal diseases that require new treatments. We investigated the neutralization of cholera toxin by five plant extracts obtained from the Rosaceae family that have been traditionally used in Poland to treat diarrhea (of unknown origin).

**Methods:**

Hot water extracts were prepared from the dried plant materials and lyophilized before phytochemical analysis and assessment of antimicrobial activity using microdilution assays. The ability of the plant extracts to neutralize cholera toxin was analyzed by measurement of cAMP levels in cell cultures, enzyme-linked immunosorbent assay and electrophoresis, as well as flow cytometry and fluorescence microscopy studies of fluorescent-labeled cholera toxins with cultured human fibroblasts.

**Results:**

The antimicrobial assays displayed modest bacteriostatic potentials. We found that the plant extracts modulate the effects of cholera toxin on intracellular cAMP levels. Three plant extracts (*Agrimonia eupatoria* L., *Rubus fruticosus* L.*, Fragaria vesca* L.) suppressed the binding of subunit B of cholera toxin to the cell surface and immobilized ganglioside GM_1_ while two others (*Rubus idaeus* L.*, Rosa.canina* L.) interfered with the toxin internalization process.

**Conclusions:**

The traditional application of the Rosaceae plant infusions for diarrhea appears relevant to cholera, slowing the growth of pathogenic bacteria and either inhibiting the binding of cholera toxin to receptors or blocking toxin internalization. The analyzed plant extracts are potential complements to standard antibiotic treatment and Oral Rehydration Therapy for the treatment of cholera.

**Electronic supplementary material:**

The online version of this article (10.1186/s12906-019-2540-6) contains supplementary material, which is available to authorized users.

## Background

Diarrhea causes millions of deaths each year as a result of the action of a wide range of pathogens, including enterotoxin-producing strains of bacteria such as *Escherichia coli* or *Vibrio cholerae* [1]. The pathogens are mainly spread by water or food contaminated with human or animal feces, and from person to person. The *V. cholerae* infections result in severe diarrhea that, without proper treatment, can kill within a few hours. Children and the elderly in developing countries constitute the largest groups of fatalities. Cholera outbreaks are related to poor sanitation and usually occur after cataclysm or during a war when access to clean water is limited. The most recent cholera outbreak started in October 2016 in Yemen. Over 8 months, 101,820 cases of cholera were registered with 791 deaths [2]. In developed countries, only sporadic, often imported cases of *V. cholerae* infection occur [3–5] but diarrhea from pathogenic *E. coli* strains producing similar toxins regularly result in thousands of hospital patients, for example a 2011 outbreak affecting Germany and its’ neighboring countries also resulted in 50 deaths [6]. The *E. coli* infections are usually not as dangerous as cholera but still cause many problems, especially for travelers, children and the elderly [1]. The main virulence factor of the above-mentioned bacteria is the production of toxins belonging to the AB_5_ family, such as cholera (CTX) or heat-labile enterotoxins (LT and LT-II). The toxins are expressed and secreted as a response to bacterial quorum sensing once a colony has reached a mature size [7]. Structurally, the AB_5_ toxins contain a catalytic subunit A_1_ linked by a short A_2_ peptide to a pentameric subunit B that binds to gangliosides located on human cell surfaces [8]. After secretion from the bacteria, the toxin binds to gangliosides and is then internalized from the plasma membrane by endocytosis and undergoes retrograde trafficking through the trans-Golgi network to the lumen of the endoplasmic reticulum. Here, subunit A is dissociated from the holotoxin, refolded and released to the cytoplasm where it causes constitutive activation of adenylate cyclase, resulting in activation and the conversion of ATP to cAMP. The high concentration of cAMP results in the opening of cAMP-dependent chloride channels and secretion of chloride ions into the lumen of the small intestine. Accumulation of chloride causes secretion of sodium ions into the lumen of the small intestine across the tight junction. An increased concentration of sodium chloride in the small intestine lumen creates an osmotic gradient that results in water outflow into the small intestine lumen across the tight junction [9]. This is the point when diarrheal symptoms start. Table 1 gives a list of different stages of *V. cholerae* infection where a pharmaceutical intervention may provide relief of cholera symptoms/infection. Each stage involves a number of potential molecular targets. It is possible to block the multi-step mechanism of action of cholera toxin at several different stages. Toxin neutralization after release by the bacteria and before internalization by human cells is one of the most accessible targets for a natural remedy or functional food to act on, and these stages are highlighted in bold in Table 1. Targeting a protein in human cells (italics, Table 1) is more challenging and more difficult to gain approval while the targeting of bacteria (normal text, Table 1) raises questions due to the growing knowledge of the importance of the diversity and health of the gut microbiome.

**Table 1:** Stages of *V. cholerae* infection

Stage/Action^a^	Test methods	Active plant extracts	reference
1. Initial anchoring	Anti-adherence Anti-invasion	*Aegle marmelos*	[10]
2. Stress induction	Luminescent assay	Several plant extracts, including *Rosmarinus officinalis*	[11]
3. Bacteriostatic	Various	Various	e.g. [10, 12–14]
4. Bacteriocidal			
5. Quorum sensing	Native bioluminescence	Small molecule screen	[15]
6. CTX expression and secretion	rtPCR ELISA of Cell free culture supernatant	Capsaicin Several plants	[16] [17]
7. CTX disassembly	**PAGE**	**(−)-Epicatechin from** ***Chiranthodendron pentadactylon***	**[19]**
8. CTX aggregation	**Centrifugation, PAGE**	**Plant derived polyphenols**	[18]
9. Inhibition of GM_1_ binding	**GM**_**1**_ **ELISA**	**Screening of 297 Chinese herbs**	[19]
*10. Intracellular trafficking*	*Biotinlabeled CTX based assay*	*Resveratrol - found in red grapes, berries and peanuts*	[20]
*11. CTA inhibitor*			
*12. Channel blocker*	*Patch clamp*	*Mixture including agarwood and clove*	[21]
99. Taste^b^	Patient based	apple	[22]

^a^The stages can be grouped based on bacterial target (normal text), extracellular toxin target (bold) and human cell target (italics)

^b^The taste of ORT might be an important factor for the treatment of under-fives

Various plant species are traditionally used by many societies to alleviate and cure diarrhea. The properties of traditional plant extracts are worth exploring as they can stop or kill bacterial growth, neutralize or deactivate enterotoxins, or provide useful microelements and vitamins [23]. Over the years, the healing properties of many plants have been superseded by synthetic pharmaceuticals, often derived from the active constituents of plants. Bacterial pathogens are becoming more resistant to commonly applied antibiotics and it is important to find new sources of antibacterial agents. However, this is unattractive in the case of cholera due to the development costs of a new drug and the fact that the disease can develop rapidly in a patient. Plant extracts do not provide the power of modern antibiotics. Instead, plant extracts may provide an attenuation of the many steps in the *V. cholerae* infection lifecycle, including the latent, early stages of the disease, moderate the effects of diarrhea and lead to improved survival and recovery rates. The citations given in Table 1 are to works describing the methodology to test plant extracts at each stage of the cholera infection, with the exception of the quorum sensing stage that used a small molecule library. If we can identify plant extracts that act at different stages of the *V. cholerae* infection lifecycle then it is possible that mixtures of plant extracts will provide a synergistic effect on the infected population, as well as individuals. This may help patients at different stages of infection, including asymptomatic carriers. The range of active metabolites produced by plants potentially treats a broad range of pathogenic strains and it will be more difficult for bacteria to develop resistance than with modern antibiotics. Furthermore, plant extracts based on accepted traditional medicines or functional foods will be more quickly, and cheaply, developed and applied.

In this study, we focus on the anti-enterotoxic activities of common European species belonging to the *Rosaceae* family: *Agrimonia eupatoria* L. (common agrimony), *Fragaria vesca* L. (wild strawberry)*, Rubus fruticosus* L. (blackberry)*, Rubus idaeus* L. (raspberry) and *Rosa canina* L. (rose), which for centuries were used in Poland as natural medicines for diarrhea [24–28]. The recommended doses, methods of infusion preparation and references are listed in Table 2. Neither the pathogenic target of these herbs is known, nor their mechanism of action. However, Poland suffered from regular cholera outbreaks in the 19th and early 20th centuries [29], and sporadic cases are still reported with non-O1 *V. cholerae* strains found in contaminated bodies of water [5]. Therefore, in this study, we wanted to analyze if the above-mentioned plant extracts have antimicrobial activities and/or can neutralize cholera toxin binding to receptors. In doing so, we obtained some positive results and also found that assays employing CTX gave less positive results than CTB.

**Table 2:** Traditional preparation of plant infusions for the treatment of diarrhea in Poland

Plant	Common Name	Used part	dry material weight [g]	Added water [mL]	Concentration (g) / liter^a^	Infusion preparation	Daily oral doses	References
*Agrimonia eupatoria* L.	agrimony	aerial parts	1.5–4	≤ 250	6–16	The stated amount of boiling water is poured over the dry plant powder, covered and incubated for 15 min to 8 h	2–3	[26, 27]
*Rubus fruticosus* L.	blackberry	leaves	1–2	250	4–8		3^b^	[28]
*Rubus idaeus* L.	raspberry	leaves	1.5–8	150	10–53.3		2–3	[25, 27]
*Rosa canina* L.	rosehip	fruit	2–5	150	13.3–33.3		≥ 3	[24, 27]
*Fragaria vesca* L.	wild strawberry	leaves	1–2	250	4–8		3^b^	[27, 28]

^a^Equivalent amount of dry plant material per liter of hot water to produce a traditional preparation

^b^Recommended dose for children is 80–125 mL

## Methods

### Plant material and extraction

The plants were dried from their natural state, and cut or chopped. All plant materials were authenticated and tested to comply with British and European food/pharmacopeia standards by the supplier, Bristol Botanicals Limited (Bristol, UK). The list of the used species and batch numbers are presented in Table 3. The aqueous extracts were prepared by pouring 25 mL of boiling Milli-Q (MQ) grade water onto 1 g of plant material, allowed to cool to room temperature, and left to stand for over 18 h. The extracts were decanted and passed consecutively through filter paper and 0.4 μm cellulose acetate filters (Whatman, UK). To further minimize contamination or degradation, the plant extracts were frozen and lyophilized to dryness, after which the extraction yields were calculated (Table 3), and then stored at − 20 °C. The lyophilized materials gave consistent data over several months.

**Table 3:** Yields of prepared plant extracts

Latin Name	Common Name	Used part	Batch number	% yield	Concentration (g)/ liter^a^	cytotoxicity (IC 50) [mg/mL]
*Agrimonia eupatoria* L.	agrimony	aerial parts	MHS002/ 110,324	12.6	19.8	5
*Rubus fruticosus* L.	blackberry	leaves	MHS016/ 100,621	14.2	17.6	2.5
*Rubus idaeus* L.	raspberry	leaves	MHS121/ 177,905	19.4	12.9	2.5
*Rosa canina* L.	rosehip	rosehip (fine cut)	MHS125/ 7640	29.5	8.5	> 10
*Fragaria vesca* L.	wild strawberry	leaves	MHS144/ 184,439	11.3	22.1	2.5

^a^equivalent amount of dry plant material per liter of hot water to produce a 2.5 mg/mL plant extract concentration

### Mammalian cell culture

Primary human skin fibroblasts (line C688) were obtained from the Department of Metabolic Diseases, The Children’s Memorial Health Institute in Warsaw, Poland [30]. Ganglioside GM_1_ molecules, receptors for CTX, are one component of fibroblast membranes. C688 cells were cultured in Dulbecco’s Modified Eagle’s Medium (DMEM) (Sigma, USA) with 10% fetal bovine serum (FBS, Gibco, South America), 100 units/mL penicillin and 100 μg/mL streptomycin (Sigma, USA) at 37 °C in a humidified atmosphere containing 5% CO_2_, on 100 mm *tissue-culture treated dishes* (Corning BD, USA) until 80–90% confluence. To dissociate the adherent cells, dishes were rinsed with phosphate buffered saline solution pH 7.5 (PBS, 10 mM Na_2_HPO_4_, 1.76 mM KH_2_PO_4_, 2.7 mM KCl, 136 mM NaCl) and incubated with 2 mL (0.5 mg/mL) of porcine trypsin (Sigma, USA) for 7 min at 37 °C. Cells were collected by centrifugation (500 g for 3 min), resuspended in fresh medium and counted under a light microscope (Zeiss Observer Z1, Germany) using a Bürker chamber.

### Cytotoxic effect of plant extracts (MTT method)

The cytotoxic activity of plant extracts was determined using the standard MTT method (according to a Sigma protocol). We seeded 5 × 10^3^ C688 cells/well into 96 well plates and cultured them as described above. The next day, the medium was discarded and cells were treated with 200 μL of several different concentrations of plant extracts diluted in DMEM with 1% FBS (range 5 to 0.078 mg/mL). After 24 h incubation at 37 °C, the medium was discarded and wells were washed twice in PBS, pH 7.5. To determine cell viability, 20 μL of 5 mg/mL MTT ((3-[4,5-dimethylthiazol-2-yl]-2,5-diphenyltetrazolium bromide), Sigma, USA) in PBS and 180 μL of DMEM without FBS were added to the wells and incubated for 4 h at 37 °C. Then, the medium was carefully discarded and 200 μL of 40 mM HCl in isopropanol was added. After 10 min incubation, the absorbance was measured at 570 nm with the background wavelength set to 630 nm using a SpectraMax M5^e^ plate reader. All experiments were carried out in triplicate. The percentage of viable cells was calculated using Eq. 1:

1 $$ \%\mathrm{viable}\ \mathrm{cells}={\mathrm{A}}_1\ \mathrm{X}\ 100\%/{\mathrm{A}}_0 $$

where A_0_ is the value of the absorbance for control conditions (which was considered 100%) and A_1_ is the value of the absorbance for tested samples, reduced by the value of background.

### Microbial strains

The aqueous plant extracts were tested against four bacterial strains: *Escherichia coli* ATCC 25922, *E. coli* O44 (834/04, collection of National Medicine Institute, Warsaw, Poland), *Vibrio cholerae* O395-tacCTB strain (Chiron Srl./Novartis) and *Lactobacillus rhamnosus* (ATCC 53103).

### Broth microdilution assay

The antimicrobial activity of plant extracts was determined by a standard microdilution technique using 96-well microtiter plates. Bacterial inoculates were prepared from 12 h liquid cultures grown on Mueller-Hinton (MH) broth. The plant extracts were dissolved in MH medium to a concentration of 2.5 mg/mL and a series of six, two-fold dilutions of plant extracts in MH broth (range 2.5–0.078 mg/mL) were prepared across the microtiter plates. To each well, 10 μL of inoculum (~ 0.2 × 10^5^ bacterial cells / well) was added. The wells were filled with MH broth to 200 μL total volume. As a negative control, plant extracts were replaced with MH broth and as a positive control – bacteria were incubated with 35 μg/mL of chloramphenicol. After 18 h incubation at 37 °C, the absorbance of each well was measured at 600 nm using a SpectraMax M5^e^ plate reader. The bacterial growth was calculated with Eq. 2:

2 $$ \%\mathrm{bacterial}\ \mathrm{growth}={\mathrm{A}}_1\ \mathrm{X}\ 100\%/{\mathrm{A}}_0 $$

where A_1_ = sample absorbance, A_0_ = absorbance of negative control.

Using this scale, 0% is total bacterial growth inhibition and 100% is the bacterial growth in the absence of plant extracts. The plant extract concentration that resulted in a bacterial growth reduction by more than 80% was interpreted as a minimal inhibitory concentration (MIC), while a reduction by more than 50% was defined as a concentration that limited bacterial growth.

To determine the minimal bactericidal concentrations (MBC) of the plant extracts, 2 μL of suspension after 18 h culture was applied to MH agar plates and cultured at 37 °C for 18 h. The lowest concentration of plant extract without bacterial colonies was interpreted as the MBC. The experiments were performed in triplicate.

### cAMP assay

The cAMP levels in cultured C688 cells were determined using a cAMP-Glo Max Assay (Promega, USA), according to the manufacturer’s protocol. Cells were grown in tissue culture treated 96-well, white plates with clear, flat bottoms (Brand, Germany). To each well, 10^3^ C688 cells were added and cultured overnight under standard conditions. The next day, the medium was discarded and a mixture of 2.5 mg/mL plant extract or gallic acid and 25 nM CTX (2.125 μg/mL) in DMEM supplemented with 30 mM MgCl_2_ was added to each well. The plates were incubated for 2 h at 37 °C in 5% CO_2_. Next, the proper amount of detection solution and kinase glo reagent were added. The luminescence (RLU) was measured using a SpectraMax M5^e^ plate reader. As controls, cells were incubated with only: plant extracts, CTX, or DMEM. The cAMP level was calculated as a change in RLU (ΔRLU) for the sample incubated with only plant extract/gallic acid/DMEM and the sample incubated with a mixture of plant extract/gallic acid/DMEM and CTX. Each sample was tested in three repeats.

### Ganglioside GM_1_- CTX binding assay

The ganglioside GM_1_- CTX interaction was analyzed according to a published protocol based on the ELISA method ([18], with modification). The 96-well, clear, flat-bottom immune-plates (Nunc, Denmark) were coated with 25 ng ganglioside GM_1_ resuspended in 50 μL ethanol and incubated at 37 °C for 2 h, to dryness. The wells were washed three times with 200 μL wash buffer (0.05% Tween 20 in PBS, pH 7.5), blocked with 200 μL blocking buffer (0.5% bovine serum albumin (BSA, Sigma, USA) in PBS) for 18 h at 4 °C, and washed three more times with 200 μL wash buffer. Wells coated with ganglioside GM_1_ were incubated with 2.5, 1.25, 0.625, 0.3, 0.15, 0.075 mg/mL of plant extracts and 0.25 μg/mL CTX for 2 h, in a total volume 200 μL. As negative controls, three wells coated with GM_1_ were incubated with CTX to provide maximal measurements or with 2.5 mg/mL plant extract to provide baseline measurements. To prepare a positive control, the binding sites of CTB were blocked with free GM_1_ as an inhibitor, by pre-incubating CTX with GM_1_ for 1 h. The resulting, inactivated CTX was added to wells coated with GM_1_ and incubated as described above. The wells were washed three times with 200 μL of wash buffer followed by incubation with 50 μL of anti-CTB antibody (Invitrogen), diluted 1:4000 in 0.5% bovine serum albumin (BSA), for 90 min at room temperature. After triple washing, wells were incubated for 75 min at room temperature with 50 μL of secondary anti-mouse antibody conjugated with horse radish peroxidase (Sigma), diluted 1:15,000 in 0.5% BSA. Wells were washed three times with wash buffer, once with PBS and then dried. To visualize the CTX bound to GM_1_, 100 μL 3,3′,5,5′-Tetramethylbenzidine (Millipore, USA) was added to each well and incubated for 15 min. To stop the reaction, 100 μL of stop solution (0.5 M H_2_SO_4_) was added and the absorbance was measured at 450 nm using a SpectraMax M5^e^ plate reader. Each assay was repeated six times.

### Ganglioside GM_1_ and CTB-FITC binding assay

The protocol to this method is similar as for CTX (above). Instead of unlabeled CTX, 1.25 μg/mL of CTB-FITC (Sigma, Israel), CTB labeled with a fluorescent fluorescein derivative, was incubated with the appropriate concentration of plant extracts (2.5–0.075 mg/m, serial dilutions) in a total volume of 200 μL. After 2 h incubation at room temperature, wells were washed three times with PBS. Next, 200 μL of PBS was added and the intensity of fluorescence was measured at 490 nm excitation and 525 nm emission, using an Infinite M1000 PRO plate reader (Tecan). Each assay was repeated six times.

### Fluorescence activated cell sorting (FACS) assay: quantitative assay

This assay was performed to analyze the ability of plant extracts to inhibit the binding of CTB-FITC to ganglioside GM_1_ naturally embedded in the extracellular surfaces of fibroblasts (C688). C688 cells (5 × 10^4^) were re-suspended in DMEM and incubated for 60 min at 37 °C with 2.5 mg plant extract and 0.25 μg CTB-FITC in 1 mL total volume. As controls, we used: (i) cells exposed only to toxin, which was treated as a negative control, (ii) cells exposed only to plant extract, which allowed us to account for autofluorescence from the plant extract and (iii) cells treated with inactivated FITC-CTB, obtained by pre-incubation of 0.5 μg/mL GM_1_ and 0.25 μg/mL FITC-CTB for 1 h, which was treated as a positive control*.* To all samples, 50 μg/mL propidium iodide (PI, Sigma, USA) was added to determine cell viability. The number of stained cells and the intensity of the fluorescence of 10^4^ cells were analyzed by flow cytometry (FACSCalibur, Becton Dickinson, USA) in two channels: FL-1 (green) for FITC and FL-3 (red) for PI. The data were analyzed using CellQuest acquisition/analysis software. Each sample was tested in three independent assays.

### Fluorescent microscopy – qualitative assay

This assay was performed to visualize the activity of the plant extract and CTB-FITC on cells containing ganglioside GM_1_. C688 cells (5 × 10^3^) were seeded onto cover glass slips and cultured for 18 h according to the described procedure. Cells adherent to the glass were washed in PBS and incubated at 37 °C for 1 h with 5.0, 2.5 or 1.25 mg/mL plant extract and 0.25 μg/mL CTB-FITC in 500 μL DMEM, and then fixed with 3% paraformaldehyde. The nuclei were stained with 0.3 mg/mL of 2-(4-Amidinophenyl)-6-indolecarbamidine dihydrochloride (DAPI, Sigma, Israel). The cover glass slips were stuck to microscope slides using MOVIOL solution and observed under a Fluorescent Microscope (Axio Observer Z1, Zeiss). Each sample was tested in three independent repeats.

### Discontinuous polyacrylamide gel electrophoresis under denaturing conditions (SDS-PAGE)

Our protocol is based on a published method with some modifications [31]. CTX (2 μg) was incubated with 1 mg plant extracts in a twofold diluted range (1–0.0015 mg) for 1 h at room temperature. The total sample volume was 12 μL. Next, samples were applied to 10% denatured gels and, using the Tris/Tricine discontinuous electrophoresis system ([32], with modifications), run for 90 min at 90 V (15 min) and next at 130 V. The gels were washed and stained with Blue BANDit Protein Stain reagent (VWR, USA). As a negative control, we used CTX without plant extracts. To avoid false positive results and eliminate thresholds we used plant extracts without CTX. As positive controls, CTX was incubated with ganglioside GM_1_ solution (2–0.03 μg) and gallic acid (0.1–0.0015 mg). The experiments were performed in triplicate

### Statistical analyses

Data were obtained from three or six measurements, as defined in the specific sections, and were expressed as the means ± standard deviation. Statistical analyses were performed using one-way ANOVA, followed by the Bonferroni-Holm post hoc test [33]. Statistically significant differences between groups were defined as *p*-values less than 0.05 with the Bonferroni-Holm correction.

## Results

### Preparation and preliminary characterization of plant extracts

After hot water extraction and filtration, the lyophilized plant powders were stored at -20 °C. The efficiency of the extraction process is shown for each plant species in Table 3. The yield varied between 11 and 19%, but is almost 30% for rosehip. The results of the phytochemical analyses [34–36] of each aqueous extract are given in Additional file 1. The cytotoxic activity of all extracts was tested using the MTT method against C688 cells, Table 3. Concentrations above 2.5 mg/mL of raspberry leaf, blackberry leaf and wild strawberry leaf were found to be cytotoxic, for agrimony this effect is above 5 mg/mL, while the rosehip extract is not cytotoxic even at 10 mg/mL.

### Antimicrobial activity tests

**Fig. 1:**
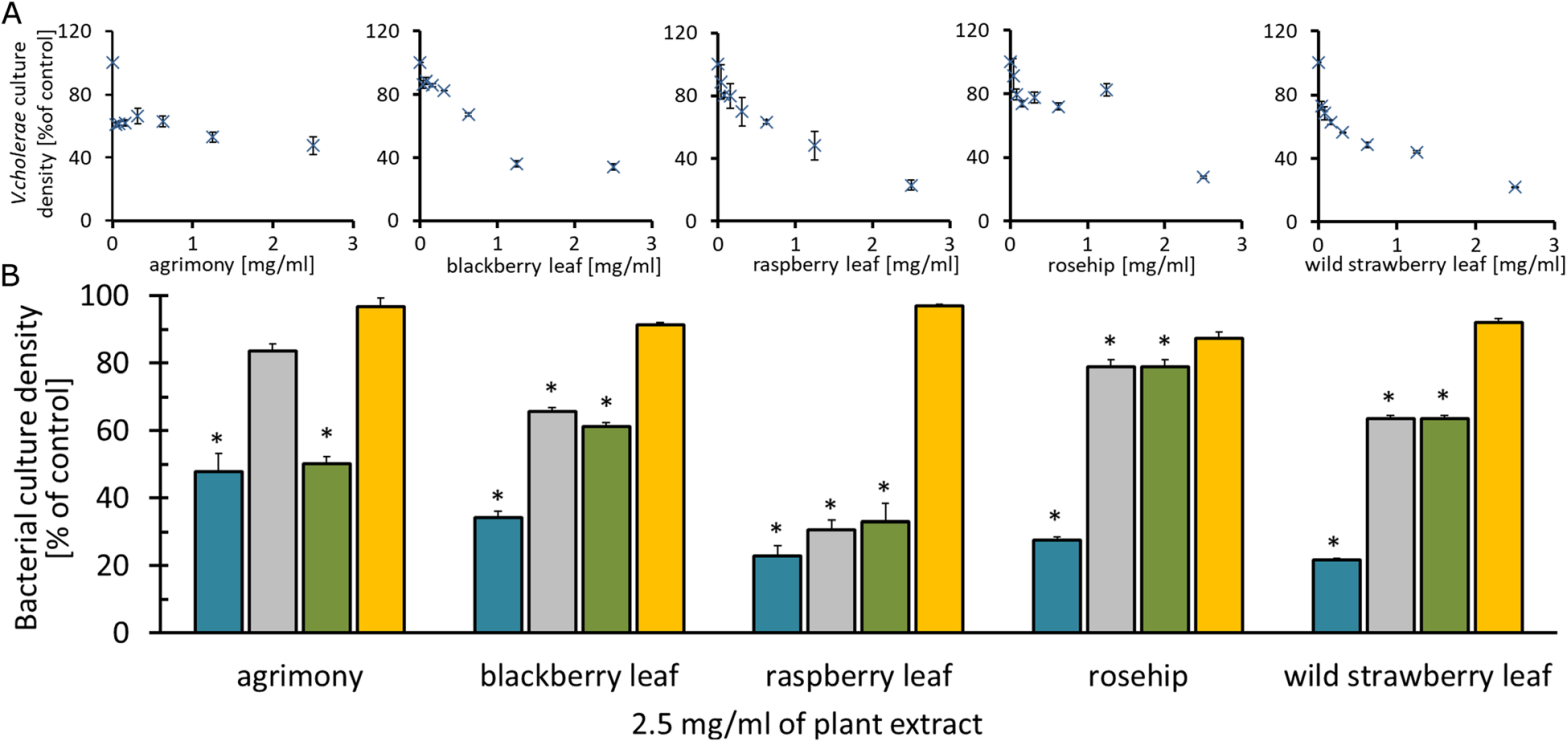
Bacteriostatic properties of plant extracts. **a** Density of 18 h *V. cholerae* cultures as a function of incubation with different plant extract concentrations (0–2.5 mg/mL); **b** Culture density after 18 h incubation of four bacterial strains: *V. cholerae* (blue), *E. coli ATCC 25922* (grey), *E. coli O44* (green) and *L. rhamnosus* (yellow) with 2.5 mg/mL aqueous extracts of plant extracts. Values are mean ± standard errors of three independent assays. * *p* < 0.05, compared with untreated bacterial cultures (100%)

The antimicrobial activity of plant extracts were analyzed using standard microdilution assays, stages 2 and 3 of Table 1. Bacteria were cultured with plant extracts in the concentration range 0.078–2.5 mg/mL, and additionally at 10 mg/mL. With an increased concentration of plant extracts, the OD_600_ of 18 h cultures decreased, which suggests that the plant extracts slow bacterial growth but did not reduce it by 80% (Fig. 1a). The 2.5 mg/mL concentrations of each extract limited bacterial growth (Fig. 1b) but did not possess bactericidal activity against *V. cholerae*. When the concentration of the plant extracts were increased to 10 mg/mL (data not shown), bactericidal activity for agrimony and blackberry leaf extracts was observed. None of the tested plant extracts, even at 10 mg/mL, inhibited the growth of *Lactobacillus rhamnosus*, which was chosen to represent beneficial gut bacteria. The 35 μg/mL of chloramphenicol, used as a positive control, has bactericidal activity for all of the tested bacteria species (it reduced the bacterial culture densities by more than 99% compared to untreated samples). Table 4 summarizes the antimicrobial results with significant data (effects at or below traditional doses) given in bold.

**Table 4:** Concentration of plant extracts to reduce bacterial culture densities by 50%

Extract	Bacterial culture^c^				Bactericidal potential^b^
	*V. cholerae* ^*a*^	*E. coli ATCC 25922* ^*a*^	*E. coli O44* ^*a*^	*L. rhamnosus*	
agrimony	**1.25**	10	2.5	> 10	No^d^
blackberry leaf	1.25	10	> 10	> 10	No^d^
raspberry leaf	**1.25**	**2.5**	**2.5**	> 10	No
rosehip	**2.5**	> 10	> 10	> 10	No
wild strawberry leaf	**0.625**	> 10	> 10	> 10	No

^a^Entries in bold indicate effects at, or below, traditional doses

^b^Tested against *V. cholerae*, *E. coli ATCC 25922*, *E. coli O44* and *L. rhamnosus*

^c^Values in mg/mL applied plant extract

^d^Bactericidal potential observed at 10 mg/mL levels, above Traditional concentrations

### Aqueous plant extracts reduce cAMP production

**Fig. 2:**
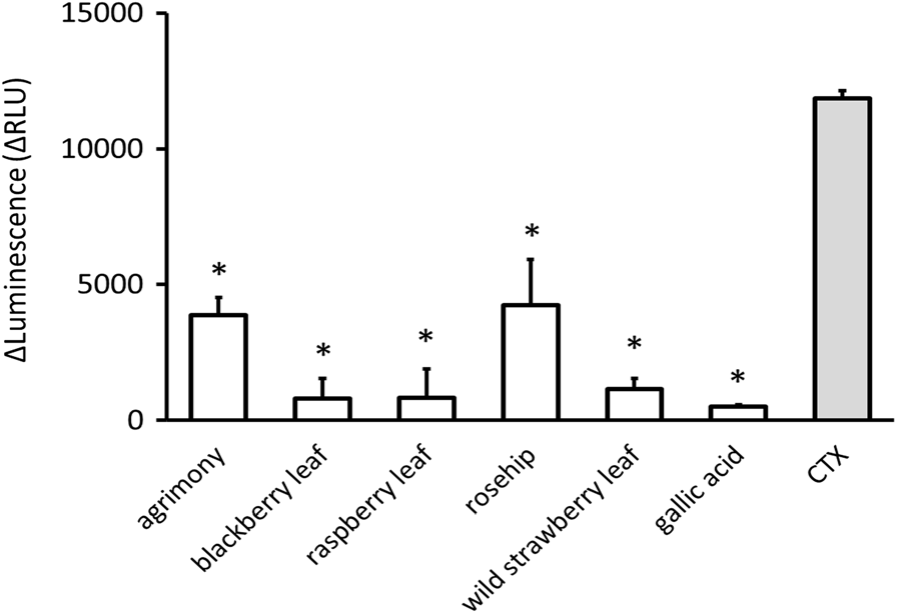
Plant extracts modulate the increased cAMP concentrations caused by the addition of cholera toxin to cell cultures. Bioluminescent assay showing the differences (ΔRLU) between cAMP production by fibroblasts treated only with plant extracts (2.5 mg/mL agrimony, blackberry leaf, raspberry leaf, wild strawberry leaf, gallic acid and 10 mg/mL rosehip) and fibroblasts treated with plant extracts and CTX (25 nM) (white). As a control, the difference between untreated and CTX treated cells is shown (grey). Values are mean ± standard error of three independent assays. * *p* < 0.0023, compared with control

The changes of cAMP levels in cell cultures were determined using the cAMP-Glo™ Max Assay from Promega. This assay reports the cumulative effect of the plant extracts on stages 7–10 of Table 1, as well as other possible intracellular effects not specified here. Treating cell cultures with cholera toxin resulted in larger cAMP concentrations in the cell, which then lead to a reduction in the luminescence intensity (RLU). The difference in luminescence (ΔRLU) between untreated and cholera toxin treated cells was around 12,000 RLU, and this served as a control for plant extract or gallic acid treated samples (Fig. 2). The application of 2.5 mg/mL agrimony extract caused the reduction of cAMP levels by 70% compared to the control, while application of the same concentration of wild strawberry leaf, blackberry leaf, raspberry leaf and gallic acid reduced it by more than 90%. The incubation with 10 mg/mL rosehip extract reduced the cAMP level by 65%. These positive results indicate that the tested plant extracts interfere with the mechanism of action of cholera toxin to different extents and we decided to employ further tests to probe the roles of the different plant extracts.

### Aqueous plant extracts inhibit the binding of CTX and CTB to ganglioside GM_1_

**Fig. 3:**
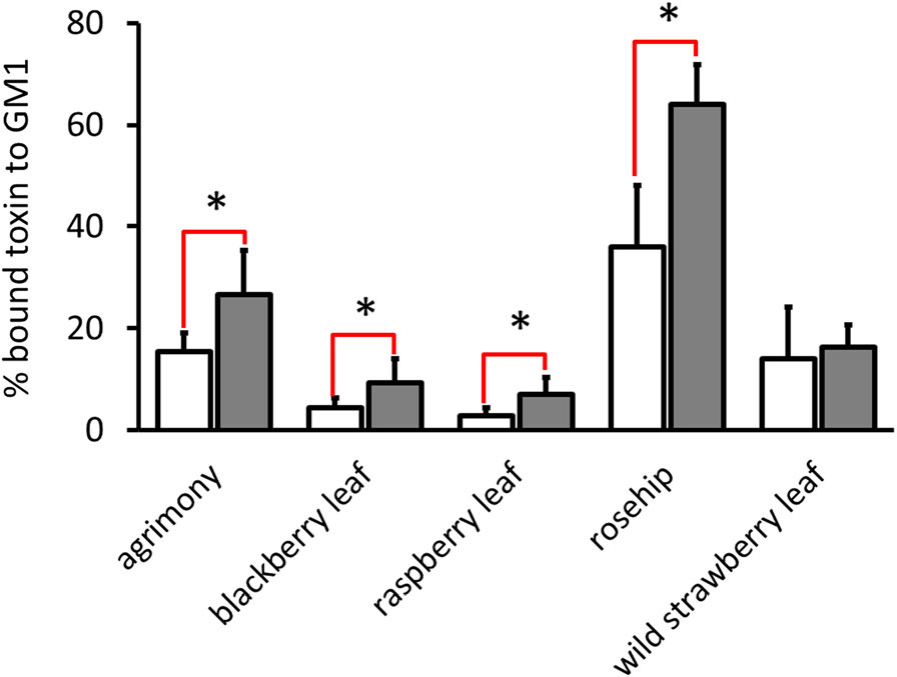
Plant extract interference in the cholera toxin recognition of GM_1_ receptors. Comparison of 2.5 mg/mL plant extract activities preventing the binding of subunit B of cholera toxin (white) and cholera toxin (gray) to immobilized GM_1_. Values are means ± standard errors of six independent assays, *p* < 0.01, compared with untreated CTX or CTB bound to receptors (100%) for all data; * *p* < 0.01, for differential response of a plant extract on the binding of CTX or CTB to GM_1_

**Fig. 4:**
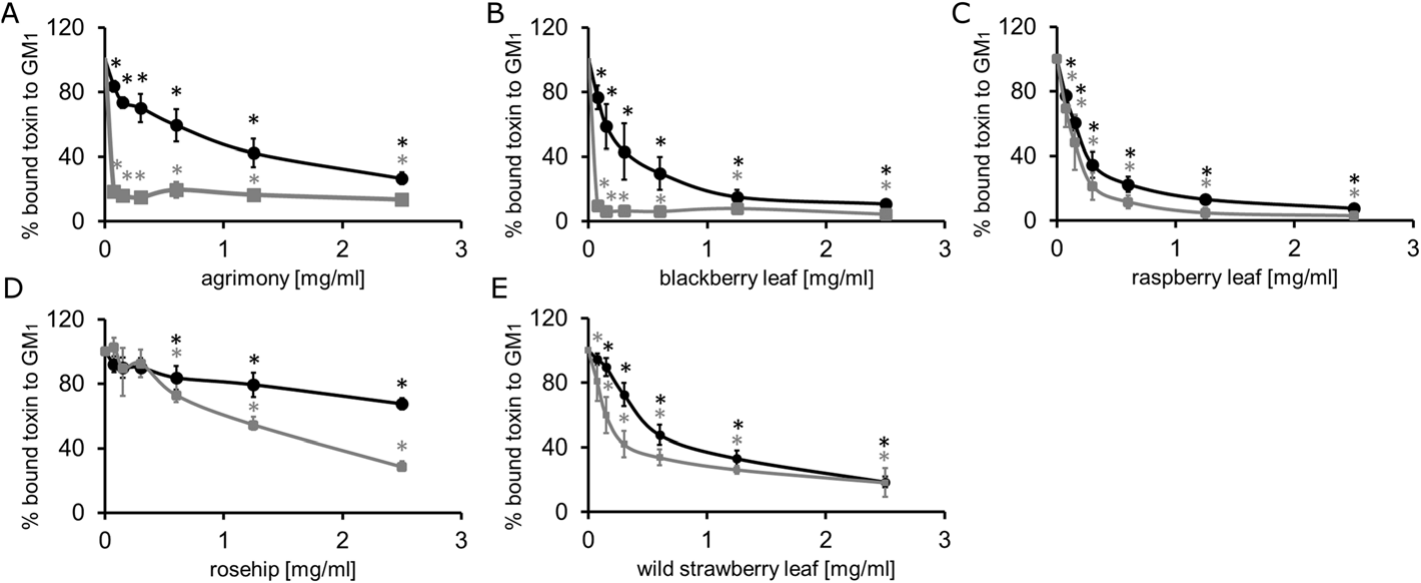
Plant extract effects on the prevention of binding cholera toxin to immobilized GM_1_. Modified ELISA binding assay showing the amount of CTX (black) and CTB (grey) immobilized on GM_1_-coated microplates as a function of the 2.5–0 mg/mL aqueous plant extracts: **a** agrimony, **b** blackberry leaf, **c** raspberry leaf, **d** rosehip, **e** wild strawberry leaf. Values are mean ± standard error of six independent assays. * *p* < 0.01, compared with CTX or CTB (100%)

We next investigated if plant extracts inhibit the binding of cholera toxin to immobilized GM_1_ receptors, stages 8–9 of Table 1. The inhibitory ability of *Rosaceae* extracts on the binding of CTB and CTX to ganglioside GM_1_ was evaluated by competitive GM_1_-ELISA, Figs. 3 and 4. The amount of CTX and CTB bound to GM_1_ in the presence of a fixed concentration (2.5 mg/mL) of plant extract is shown in Fig. 3 while the effect of variable plant extract concentrations on toxin binding to GM_1_ is given in Fig. 4 and Table 5. Each plant extract inhibits the binding of toxin to GM_1_ but the efficiency varies among plant extracts. The weakest suppression of the binding of toxin to GM_1_ was found to be rosehip extract (Fig. 4d). Incubation with 2.5 mg/mL of rosehip extract resulted in a 68% reduction in the binding of CTB but only a more modest 28% for CTX to the immobilized GM_1_. The minimal plant extract concentration necessary to inhibit 50% of the CTX bound to the GM_1_ for blackberry leaf (Fig. 4b) and raspberry leaf (Fig. 4c) was found to be 0.15 mg/mL, while for agrimony (Fig. 4a) and wild strawberry leaf (Fig. 4e) it was found to be 0.3 mg/mL

**Table 5:** Summary of beneficial plant extract effects^a, b^

extract	Blocking of CTX binding to GM_1_ (ELISA)^c;d^	Blocking of CTB binding to GM_1_ (ELISA)^c;d^	Blocking of CTB binding (cell assay)	Blocking of CTB internalization (cell assay)^c^	Level of cAMP after CTX and plant extract treating^e^	Interaction with CTX by SDS-PAGE^f^	Pass^g^
agrimony	**0.64**	**< 0.08**	**1.25**	**1.25**	**33**	**aggregation**	Yes
blackberry leaf	**0.32**	**< 0.08**	2.5	**1.25**	**6.7**	**aggregation**	Yes
raspberry leaf	**0.32**	**0.16**	**No**	**5.0**	**7.1**	**aggregation**	Yes
rosehip	> 2.5^g^	**1.25**	**No**	**10**	**35.8**	**dissociation**	Yes
wild strawberry leaf	**0.64**	**0.32**	2.5	**1.25**	**9.7**	**aggregation**	Yes

^a^Entries in bold indicate positive effects at, or below, traditional doses

^b^At least 50% Inhibition of harmful bacteria growth using traditional extract concentrations but no inhibition of beneficial *L. rhamnosus*

^c^Values in mg/mL applied plant extract

^d^Inhibition at least 50% toxin bound to ganglioside GM_1_

^e^Values in % compared to untreated cells (100%)

^f^Effect seen at 1.3 or 2.6 mg/mL concentration of plant extract

^g^May attain 50% inhibition of toxin with traditional doses in the 4–10 mg/mL range

### Aqueous plant extracts prevent CTB-binding to human fibroblasts

**Fig. 5:**
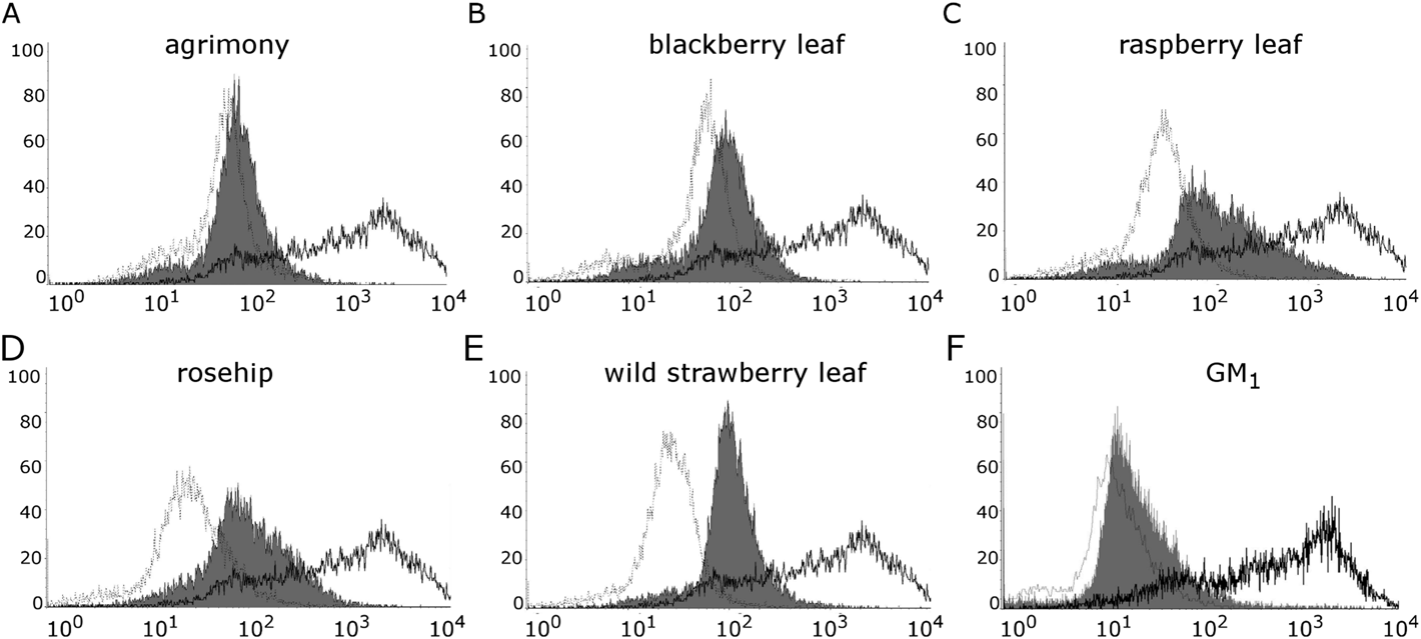
Plant extract effects on the prevention of binding cholera toxin to cellular receptors. Flow cytometry assay showing the degree of labeled cells C688 after incubation with a mixture of 2.5 mg/mL plant extract and 0.25 μg/mL FITC-CTB, where the black trace represent cells treated only with CTB-FITC, and grey trace – cells treated only with plant extract and filled grey – cells treated with CTB-FITC and plant extract: **a** agrimony, **b** blackberry leaf, **c** raspberry leaf, **d** rosehip, **e** wild strawberry leaf and **f** GM_1_

**Fig. 6:**
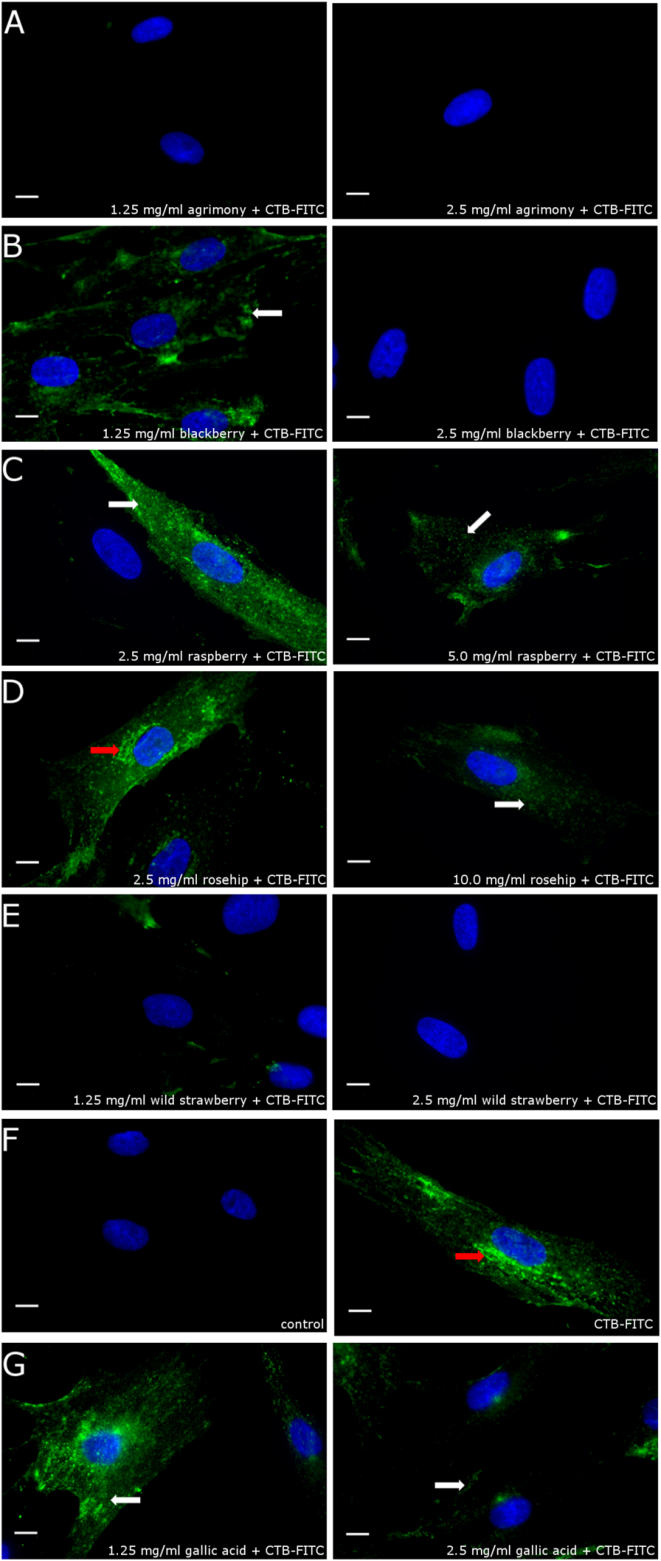
Plant extract effects on the prevention of binding cholera toxin to cellular receptors or its internalization. Fluorescent microscope assay showing the CTB-FITC (green) labeling of fibroblasts after incubation with 2.5 and 1.25 mg/mL of: **a** agrimony, **b** blackberry leaf, **c** raspberry leaf, **d** rosehip; 2.5 and 10 mg/mL, and **e** wild strawberry leaf; 2.5 and 5.0 mg/mL . As controls **f**, we used (1) untreated cells and (2) incubated only with CTB FITC and **g** incubated with 1.25 or 2.5 mg/mL gallic acid, and CTB-FITC. The nuclei of the analyzed cells were stained by DAPI (blue). The red arrows show toxin accumulated in the Golgi apparatus (see Additional file 2 for co-localization with a Golgi marker), while white arrows show toxin bound to the cell surface. Scale bar is 10 μm

CTB conjugated with a fluorophore is commonly used as a marker for lipid rafts, which are rich in gangliosides [37–39]. Exploiting this fact, the addition of CTB-FITC to human fibroblasts led to a strong fluorescent labeling of human C688 fibroblast cells, Figs. 5 and 6. Fluorescence cytometry gives a measure of CTB-FITC labeling of C688 cells in the presence and absence of plant extracts (Fig. 5). This assay also tests stages 8 and 9 of Table 1. The addition of CTB-FITC resulted in a distinct change in the fluorescence labeling of C688 cells compared to the normalized background readings. A significant lowering of the number of CTB-FITC labeled C688 cells was observed after pre-incubation of CTB-FITC with the positive control, ganglioside GM_1_ (Fig. 5f). The pre-incubation of CTB-FITC with extracts of agrimony (Fig. 5a) and blackberry leaf (Fig. 5b) resulted in similar results as GM_1_. The application of raspberry leaf (Fig. 5c), rosehip (Fig. 5d) and wild strawberry leaf (Fig. 5e) extracts only partly decreased the fluorescence intensity of labeled cells. The standard DNA staining method, based on propidium iodide (PI), was used to determine the cytotoxic effect of plant extracts during this assay. The number of cells labeled with PI did not increase in the presence of any of the plant extracts, indicating that the plant extracts were not cytotoxic at the 2.5 mg/mL concentration for the fibroblast C688 cell line.

The inhibition of the CTB-FITC binding by plant extracts was observed by fluorescence microscopy (Fig. 6). C688 cells were incubated for 1 h with CTB-FITC; next they were fixed to glass slides and observed under a fluorescence microscope. In cells treated only with CTB-FITC, the CTB-FITC accumulated in the perinuclear region (Fig. 6f2, red arrow), mostly with the Golgi apparatus (Additional file 2). A similar, perinuclear localization of CTB-FITC is observed for cells incubated with rosehip at 2.5 (Fig. 6d1) and 5.0 mg/mL concentrations, which suggests that lower concentrations of this extract do not suppress CTB binding and internalization. A higher concentration of rosehip extract (10 mg/mL) decreased the amount of toxin bound to the cell surface and suppressed its accumulation in the perinuclear region (Fig. 6d2). The application of 2.5 mg/mL of agrimony (Fig. 6a2), blackberry leaf (Fig. 6b2) and wild strawberry leaf (Fig. 6e2) inhibited the CTB-FITC binding to the cell surface. Inhibition of CTB binding was also observed at 1.25 mg/mL for agrimony (Fig. 6a1). After incubation with 1.25 mg/mL of blackberry leaf (Fig. 6b1), CTB-FITC bound to the cell surface but did not accumulate in the perinuclear region. The same result was observed after incubation with 2.5 mg/mL raspberry leaf (Fig. 6, C1) and 1.25 mg/mL wild strawberry leaf (Fig. 6e1). Incubation with an increased (5 mg/mL) concentration of raspberry leaf (Fig. 6c2) extract decreased the amount of toxin bound to the cell surface.

### Interactions between cholera toxin and plant extracts revealed by SDS-PAGE

**Fig. 7:**
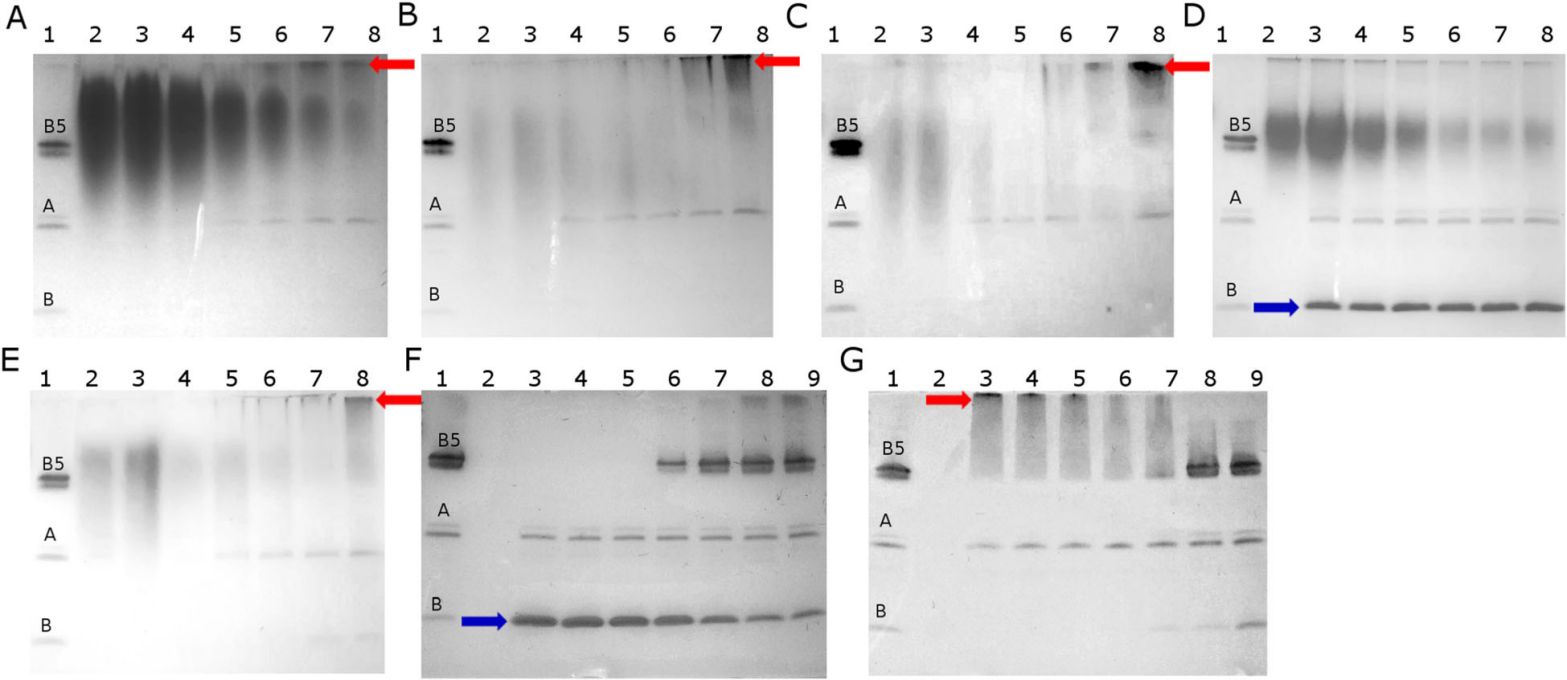
SDS PAGE analysis of the interaction between cholera toxin and plant extracts. **a** agrimony, **b** blackberry leaf, **c** raspberry leaf, **d** rosehip, **e** wild strawberry leaf, **f** gallic acid control and **g** GM_1_ control. For all panels, lane 1 contains 2 μg CTX (dissociated into: B_5_–57 kDa, A – 28 kDa and monomer B – 11.4 kDa), lane 2–1 mg of plant extracts or 0.1 mg gallic acid or 2 μg GM_1_. For panels (A-E), lanes 3–8 contain 2 μg CTX and: 1.0, 0.5, 0.25, 0.125, 0.06, 0.03 or 0.0015 mg plant extract. For panel F, lanes 3–8 contain 2 μg CTX and: 0.1, 0.05, 0.025, 0.0125, 0.006, 0.003 or 0.0015 mg gallic acid. For panel G, lanes 3–8 contain 2 μg CTX and: 2.0, 1.0, 0.5, 0.25, 0.125, 0.06, 0.03 μg GM_1_. The red arrow indicates aggregated protein, the blue arrow shows increased amount of monomer B. The reaction volume was 12 μl for all samples

We used polyacrylamide gel electrophoresis to discriminate between stages 7 and 8 of Table 1. During electrophoresis under Tris/Tricine denaturing conditions, cholera toxin is separated into a 57 kDa pentameric subunit B (B_5_), a 28 kDa subunit A and a small amount of 11.4 kDa monomer subunit B (Fig. 7, lane 1). The application of: agrimony, blackberry leaf, raspberry leaf and wild strawberry leaf extracts causes changes in the cholera toxin structure (Fig. 7a-c, e). At the highest extract concentrations, we observed the disappearance of all CTX components (lanes 3 and 4). With decreasing concentrations of plant extracts, the intensity of the subunit A band increased (lanes 5–8). The application of lower plant extract concentrations (0.06–0.0015, lanes: 6–8) resulted in the formation of aggregates (red arrow). To exclude background staining from the plant extracts, we applied samples with plant extract but without CTX (Fig. 7, lane 2).

To understand the mechanism of plant extract action, two known controls were used. The first control is based on the ability of gallic acid to inhibit cholera toxin internalization [19]. This polyphenol causes dissociation of CTB into subunit B monomers (Fig. 7f, lanes: 3–5, blue arrow). In the second variant, CTX was inactivated by ganglioside GM_1_ in solution. These receptors effectively block the ganglioside binding site of cholera toxin and cause the aggregation of subunit B, marked by the red arrow on Fig. 7g in lanes: 3–5. The intensity of the subunit A band does not depend on the GM_1_ concentration. Comparing the application of plant extracts and controls, two modes of action can be distinguished. All the plant extracts except rosehip have a mechanism of action similar to the GM_1_/CTX control where aggregation of the toxin blocks binding to the ganglioside receptors in human cells. In contrast, rosehip has a similar behavior as the gallic acid/CTX complex.

## Discussion

Oral Rehydration Therapy (ORT) is commonly used and recommended by WHO for the treatment of acute diarrhea, including cholera. This therapy is based on solutions of oral rehydration salts (ORS) containing: glucose, sodium, chloride, potassium and citrate. ORT is used to protect the organism against dehydration caused by excessive fluid losses by vomiting and watery stools, but does not act against bacteria and their toxins [40]. The incorporation of plant extracts into ORS formulae can give several potential benefits such as providing a broad range of antimicrobial and anti-toxin compounds, as well as a taste that might be better accepted by young children, Table 1. On this basis, we investigated the properties of plant extracts used in Poland to treat non-specific diarrhea.

The MTT assay and separate staining of C688 cells with PI indicated that the cytotoxicity of all the plant extracts was above 2.5 mg/mL. Assuming that the traditional preparation generates similar extraction yields, Table 3, cytotoxic effects were observed above the traditional doses for all plant extracts except rosehip. The safe concentration for rosehip is calculated as 13 g/L preparation compared with a traditional preparation of 10–53 g/L, Table 2. Overall, there appeared to be no correlation between various phytochemical analyses (phenolic or flavonoid contents, two antioxidant measures, Additional file 1) and the results of any of the antimicrobial or anti-toxin assays.

According to published data, agrimony [12], blackberry leaf [13] and raspberry leaf [13, 14] show a weak antimicrobial activity against *E. coli* strains. Our results are consistent with this data, Fig. 1. Specific, mild bacteriostatic potentials against *V. cholerae* were recorded for four of the plant extracts, Table 4, at or below concentrations used in traditional remedies. A bacteriostatic effect was measured just above the traditional preparation range for blackberry leaf. The specific growth inhibition may help beneficial bacterial colonies, for example *L. rhamnosus* that was unaffected by the plant extracts, compete with *V. cholerae* and delay the onset of diarrheal episodes. However, the bacteriostatic properties may contribute to asymptomatic carriers [41, 42]. Therefore, we cannot conclude if the bacteriostatic properties of the plant extracts are beneficial, or not, in a traditional cure of diarrhea related to cholera.

The toxic action of cholera toxin is related with the continuous production of cAMP, which leads to the opening of chloride channels and secretion of chloride anions [9]. To test if plant extracts prevent cAMP activation by CTX, the intracellular level of cAMP in cell cultures was measured. According to our data, all plant extracts suppressed the cholera toxin action on cAMP levels at levels below the traditional dose. This assay covers a broad number of stages in Table 1, as well as a number of intracellular steps for the internalization of the CTX through the endosomes, Golgi apparatus to endoplasmic reticulum and secretion to the cytoplasm. Several different assays were used to better define the action of the plant extracts.

Cell based assays (Figs. 5 and 6) utilizing CTB-FITC as a fluorescent marker provided results consistent with antibody detected CTB and CTX in the immobilized GM_1_ assays in Figs. 3 and 4, and the cAMP results (Fig. 2). Positive effects on the inhibition of CTB-FITC binding by plant extracts to C688 cells were observed. Two phenomena could be distinguished by flow cytometry and fluorescence microscopy. At higher plant extract doses, a lower labeling of C688 cells was reported by both methods. At intermediate levels, fluorescence microscopy revealed that while CTB-FITC labeled the C688 cells, the process of cellular internalization was probably blocked because CTB-FITC does not accumulate in the perinuclear region. With respect to flow cytometry data, blocking of cellular internalization would not be recognized as a positive effect of the plant extracts as the cells are still fluorescently labeled. Flow cytometry is a faster, more economical tool for screening plant extracts but may miss some beneficial plant extracts. Fluorescence microscopy adds detail to the flow cytometry data, perhaps accounting for the aggregation properties of some of the plant extracts revealed by native PAGE. High plant extract doses may fully aggregate the toxin and block the CTB/CTX recognition sites for GM_1_ receptors but intermediate plant extract concentrations may produce aggregates with remaining active GM_1_ binding sites that allow for cellular labeling. Regarding traditional levels, positive results were observed for low to mid traditional concentrations of agrimony and raspberry leaf. In the higher range of traditional concentrations, positive results were obtained for blackberry leaf and rosehip. Positive results for wild strawberry leaf were obtained only for concentrations greater than traditionally used.

Several plant extracts have been documented to interact with cholera-like toxins through different mechanisms. The Mexican plant *Chiranthodendron pentadactylon* Larrreat [31] and the Chinese plant *Chaenomeles speciosa* [43] can block the binding site of heat-labile enterotoxin produced by *E. coli* (LTX). The active compounds of these plants include gallic [19] oleanolic, ursolic and betulinic acids [43], and (−)-epicatechin [31]. These compounds bind to the toxin binding site for gangliosides (GM_1_ and others), and thus cause toxin inactivation by competition. It was also shown that plant polyphenols, such as applehenon from apples, reduced the amount of cholera toxin bound to receptors and suppressed toxin internalization, probably by toxin aggregation [18]. SDS-PAGE analysis reveals that agrimony, raspberry leaf, blackberry leaf and wild strawberry leaf extracts cause aggregation of the toxin. This mechanism appears similar as after incubation of the toxin with ganglioside GM_1_, Fig. 7. Probably, active compounds of agrimony, blackberry leaf and wild strawberry leaf bind to the cholera toxin and block the binding site or change the toxin conformation, suppressing its binding to GM_1_ in the ELISA and cell-based assays. The raspberry leaf active compounds also cause aggregation, but do not block toxin binding to the cell surface. However, according to fluorescence microscopy and cAMP data, it does inhibit toxin internalization. SDS-PAGE analysis showed that rosehip extract (at 10 mg/mL concentration) behaves like gallic acid, Fig. 7. It remains to be seen if the different effects of rosehip and the other tested extracts have a synergistic effect.

According to our data, all plant extracts inhibited CTB binding to GM_1_ more strongly than CTX. This effect might be related to the toxin structure. Possibly, when the CTA is bound to CTB, part of the CTB structure is masked by CTA against the plant extracts compound action. When the CTB is alone, it has larger, unprotected surfaces, which can be easily accessed by plant compounds that cause changes in toxin conformation and reduce the ability to bind to GM_1_. Another explanation is that the plant extract interacts with the fluorescent marker of CTB-FITC, reducing its fluorescent properties. Although we cannot define the exact mechanisms related to the different effects of CTB-FITC and CTX, our data do suggest that assays involving CTB-FITC alone may overestimate the inhibitory power of plant extracts and chemical compounds on cholera toxins.

In the context of universal ORT formulations, the plant extracts should be safe for all sections of society including children, the elderly and pregnant/lactating women, as well as people suffering with long-term diseases that are incompatible with certain foodstuffs (diabetic, allergic). Extracts of raspberry leaf, in addition to being used as traditional medicine for diarrhea, are also widely used during pregnancy. While some reports indicate that raspberry leaf extracts are safe during pregnancy [44] other reports sow doubts or caution [45, 46]. Strawberry leaf is listed as an emmenagogue in a thorough review of plant based remedies used as abortifactants, contraceptives and sterilizers [47] although the original citation [48] itself cites the emmenagogue use as being historical (Ostermann, V. (1894) La vita in Friuli, Vols. I, II. Reprinted 1974, Del Bianco Ed., Udine, Italy). While traditional medicine may have been used for several centuries, the lack of properly conducted medical studies precludes the incorporation of any of the plant extracts into a universal ORS formula without further safety studies.

The obtained results indicate that the traditional application of: agrimony, raspberry leaf, blackberry leaf, rosehip and wild strawberry leaf infusions in treating bacterial diarrhea might be effective against diseases related to AB_5_ enterotoxins, such as cholera. These plant extracts can inhibit bacterial growth, block the toxin binding to receptors, inhibit toxin internalization to the host cell and suppress cAMP overproduction (Table 5).

## Conclusions

The results described in this paper suggest that the studied plant extracts can eventually be useful complements to established ORT to improve the treatment of cholera-like diarrhea. The tested extracts do not kill bacteria, but do slow their growth, which might assist other antibiotic therapies. Simple aqueous plant extracts can inhibit the binding of cholera-like toxins to receptors or block the toxin internalization results in suppressing cAMP overproduction and chloride secretion, which protects the organism against rapid dehydration. The mechanisms involving the interactions between CTB/CTX and the studied plant extracts appear to be different, involving toxin aggregation and toxin deactivation. The mechanisms of action of these plants are still under investigation and the identification of active compounds and testing for synergistic effects is planned. We applied a number of methods and found that assays based on the safer (to use in the lab) CTB may provide a greater number of positive results than assays based on the more specific CTX, while FACS may miss some positive plant extract effects compared to the more labor intensive fluorescence microscopy method. SDS-PAGE is a useful method for examining if a plant extract can induce the ejection of CTA toxin from CTX to leave benign CTB, which may still bind to human cells without inducing diarrhea.

## Additional files

Additional file 1: Phytochemical characteristics of plant extracts (DOCX 20 kb)

Additional file 2: Co-localization of CTB-FITC and the Golgi Apparatus (DOCX 515 kb)

## Data Availability

The datasets used and/or analyzed during the current study are available from the corresponding author on reasonable request.

## Abbreviations

AB_5_: Class of enterotoxins consisting of one A subunit and 5 identical B subunits
ANOVA: Analysis of variance test
ATP: Adenosine triphosphate
BSA: Bovine serum albumin
C688: Primary human fibroblast line
cAMP: Cyclic adenosine monophosphate
CTA: Subunit A of cholera toxin
CTB: Subunit B of cholera toxin
CTX: Cholera toxin
DAPI: 2-(4-Amidinophenyl)-6-indolecarbamidine dihydrochloride
DMEM: Dulbecco’s Modified Eagle’s Medium
ELISA: Enzyme-Linked Immunosorbent Assay
FACS: *Fluorescence Activated Cell Sorting*
FBS: Fetal bovine serum
*FITC*: Fluorescein isothiocyanate
GM_1_: Ganglioside GM_1_
LT and LT-II: heat labile enterotoxins
MBC: Minimal bactericidal concentrations
MH: Mueller-Hinton broth
MQ: Milli-Q grade water (> 18 MΩ resistivity)
MTT: 3-[4,5-dimethylthiazol-2-yl]-2,5- diphenyltetrazolium bromide
OD_600_: Optical density at 600 nm
ORT: Oral Rehydration Therapy
PAGE: Polyacrylamide Gel Electrophoresis
PBS: Phosphate buffered saline
PI: *Propidium iodide*
SDS: Sodium dodecyl sulfate
