# Supplementary material for: Neutralization of cholera toxin by Rosaceae family plant extracts
Source: BMC Complement Altern Med. 2019 Jun 20;19:140. doi: 10.1186/s12906-019-2540-6 (PMC6587261; doi:10.1186/s12906-019-2540-6)
Supplement: Supplementary file 1 — Phytochemical characteristics of plant extracts (DOCX 20 kb) [file 12906_2019_2540_MOESM1_ESM.docx]

## Anti-cholera toxin potential of five Rosaceae family plant extracts

## Additional abbreviations: DPPH, 2,2-diphenyl-1-picrylhydrazyl; ECE, (-)-epicatechin equivalents; EGE, gallic acid equivalents

Additional Table S1. Phytochemical characteristics of plant extracts

| plant | Antioxidant activity | | Total polyphenols | | Total flavonoids |
| --- | --- | --- | --- | --- | --- |
|  | DPPH (IC 50) [µg/ml]^1^ | μmol Fe^2+^  in 1 mg PE | μg epicatechin  in 1 mg/ml PE | μg gallic acid  in 1 mg/ml PE | μg epicatechin  in 1 mg/ml PE |
| agrimony | 3.9 | 51.9 ± 1.9 | 229.5 ± 33.9 | 16.9 ± 2.3 | 140.9 ± 13.2 |
| blackberry leaf | 3.9 | 83.7 ± 3.1 | 313.1 ± 37.4 | 22.5 ± 2.5 | 125.4 ± 5.6 |
| raspberry leaf | 2.0 | 91.4 ± 4.2 | 365.4 ± 22.6 | 26.1 ± 1.5 | 55.2 ± 7.5 |
| rosehip | 3.9 | 38.3 ± 1.0 | 215.4 ± 55.1 | 15.9 ± 3.7 | 267.2 ± 15.8 |
| wild strawberry leaf | 7.8 | 57.0 ± 1.5 | 223.0 ± 8.5 | 16.4 ± 0.6 | 52.9 ± 8.6 |

^1^ 2,2-Diphenyl-1-picrylhydrazyl (DPPH) assay: The antioxidant activity of plant extracts was determined using the standard 2,2-Diphenyl-1-picrylhydrazyl (DPPH) radical scavenging method. To a 96-well plate, 0.5 mg/ml plant extract was added and serially diluted (the final well concentrations were 500 – 0.031 µg/ml) in a total volume 100 μl. Next, 100 μl of 1 mM methanol solution of DPPH (Aldrich, Germany) was added to each well and incubated for 30 minutes at room temperature. As a negative control, plant extracts were replaced with MQ water. As a positive control, serial dilutions of 25 µg/ml (range 25 – 0.0015μg/ml) gallic acid were used. The absorbance was measured at 517 nm using a SpectraMax M5^e^ plate reader. The results were presented as a value of plant extract concentration needed to reduce the amount of free DPPH molecules to 50 % (IC_50_). All experiments were carried out in triplicate.

^2^ Ferric reducing antioxidant power: The antioxidant activities of aqueous extracts were determined using the ferric reducing antioxidant power assay (modified to 96-well plate assay). The 50 μl of 1 mg/ml plant extract was incubated with 50 μl of 200 mM sodium phosphate buffer pH 6.6 and 50 μl of 1% (0.03 M) potassium ferricyanide (III) for 20 minutes at 50^o^C. Next, 50 μl of 10% TCA and 50 μl of 0.67% (0.04M) ferric chloride (III) were added. After 10 minutes incubation, the absorbance was measured at 700 nm using a SpectraMax M5^e^ plate reader. As a negative control, ferric chloride (III) was replaced with MQ water. As a positive control, 10 μg of ascorbic acid was used. The amount of reduced Fe(III) to Fe(II) was calculated for 1 mg plant extract using a standard curve defined by y = 0.3441x – 0.0397 (R^2^ = 0.9993), where y – absorbance at 700 nm and x – amount of Fe^2+^ (μmol) in the sample. To prepare the standard curve, serial dilutions of Fe(NH_4_)SO_4_ × 6 H_2_O solution (8 - 0.5 μmol) were used. All experiments were carried out in triplicate.

^3^ Determination of total phenolic content in aqueous plant extracts: The total phenolic content of aqueous plant extracts was determined using the commonly used Folin-Ciocalteu method. A 50 μl aliquot of 1 mg/ml plant extract (0.05 mg lyophilized powder in each sample) was mixed with 110 μl of 10-fold diluted Folin-Ciocalteu reagent (Merck, Germany) in a 96-well plate (SPL, Korea). After 10 minutes incubation, 90 μl of 0.75 M CaCO_3_ was added and the plate was incubated at room temperature, in the dark, for 2 more hours. Next, the plates were mixed and the absorbance at 750 nm was measured using a SpectraMax M5^e^ plate reader (Molecular Devices, USA). As a negative control, the plant extract was replaced with MQ water. The calibration curves were constructed using serial dilutions of 100 μg/ml (-)-epicatechin (Sigma, China) (100 - 3.125 μg/ml) and 31.125 μg/ml of gallic acid (Fluka, Switzerland) (31.125 – 0.24 μg/ml). The total polyphenol content was expressed as gallic acid (EGE) and (-)-epicatechin (ECE) equivalents in 1 mg of plant extract. The amount of polyphenols was calculated using the calibration curve calculated for (-)-epicatechin using y = 0.0353x + 0.1456 (R^2^ = 0.9807) and for gallic acid using y = 0.0787x – 0.0133 (R2 = 0.9997), where y – absorbance at 750 nm and x – amount of polyphenols (EBE or ECE; μg/mg plant material). All experiments were carried out in triplicate.

^4^ Determination of total flavonoid content in aqueous plant extracts: The total flavonoid content of aqueous plant extracts were determined in 96-well plates using aluminium chloride. A 10 μl aliquot of 20 mg/ml plant extract was mixed with 30 μl of 5 % NaNO_2_ and incubated for 5 minutes. Next, 5 μl of 10% AlCl_3_ was added. After 6 minutes incubation, the samples were neutralized with 50 μl of 4 % NaOH and 155 μl of MQ water was added and incubated for 10 minutes at room temperature. As a negative control, the plant extract was replaced with MQ water. Next, the plates were mixed and the absorbance was measured at 510 nm using a SpectraMax M5^e^ plate reader. Blank samples were prepared separately for each plant extract - AlCl_3_ was replaced with MQ water. The flavonoid content was expressed (-)-epicatechin equivalent (ECE) in 1 mg plant material. The calibration curve was constructed using serial dilutions of 50 μg/ml (-)-epicatechin (50 – 0.39 μg/ml). The total flavonoid content was calculated for 1 mg of plant extract using the calibration curve described by y = 0.006x (R^2^ = 0.9892) where y – absorbance at 510 nm and x – amount of flavonoids (ECE, μg/mg plant material). All experiments were carried out in triplicate.
