# Supplementary material for: Neutralization of cholera toxin by Rosaceae family plant extracts
Source: BMC Complement Altern Med. 2019 Jun 20;19:140. doi: 10.1186/s12906-019-2540-6 (PMC6587261; doi:10.1186/s12906-019-2540-6)
Supplement: Supplementary file 2 — Co-localization of CTB-FITC and the Golgi Apparatus (DOCX 515 kb) [file 12906_2019_2540_MOESM2_ESM.docx]

## Anti-cholera toxin potential of five Rosaceae family plant extracts


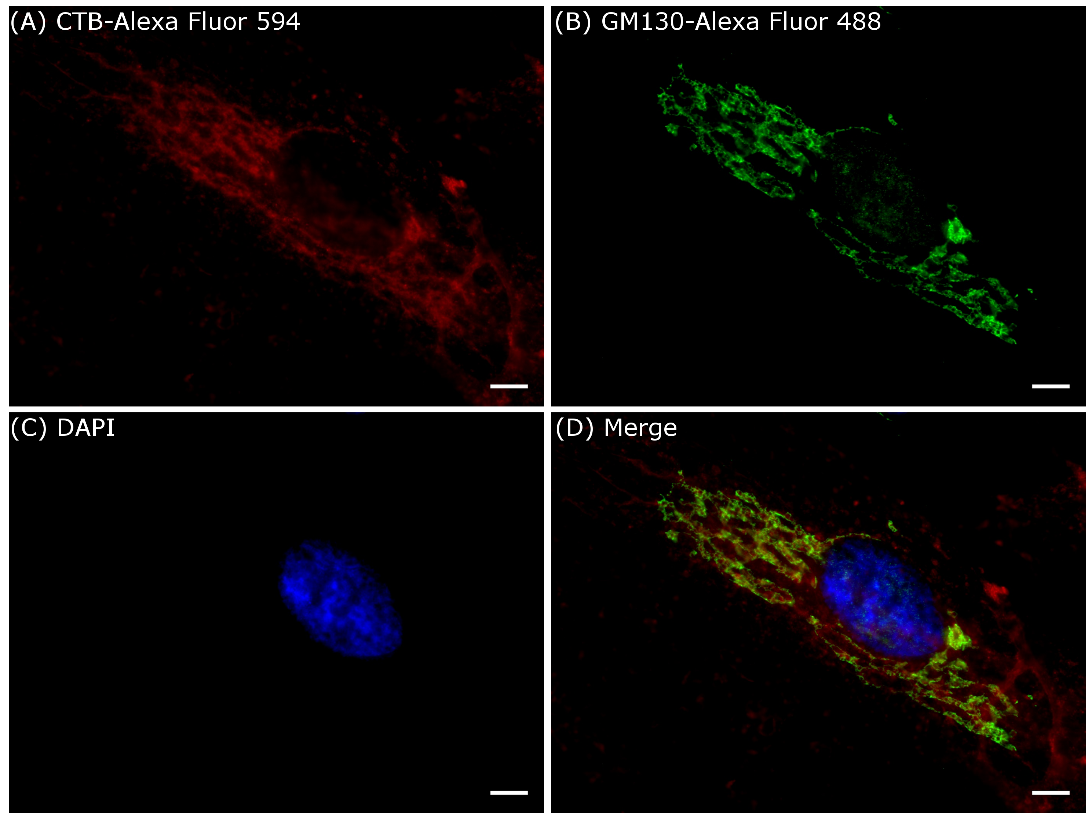


Additional File 2: Colocalization of CTB-FITC and the Golgi Apparatus. Fluorescent microscope assay showing the (A) CTB-Alexa 594 (red), (B) GM130 (Golgi, green), and (C) DAPI (nucleus, blue) labeling of fibroblast components, as well as (D) the merged image to identify colocalized molecules. Scale bar is 10 μm. The images were prepared by seeding C688 cells (5×10^3^) onto cover glass slips and culturing for 18 h according to the procedure described in the materials and methods section of the main paper. Cells adherent to the glass were washed in PBS and incubated at 37^o^C for 1 h with 0.25 μg/ml CTB-Alexa 594 in 500 μl DMEM, and then fixed with 3% paraformaldehyde. Next cells were permeabilized with 0.08% Triton X-100 for 5 min on ice and were blocked with 5 % FBS in DMEM for 1 h at room temperature. To visualize the Golgi apparatus, cells were incubated with anti-GM130 antibody (Abcam, ab52649, Golgi marker) (1:200, 0.5 FBS in PBS) for 18h at 4^o^C, followed by incubation with secondary anti-rabbit antibody conjugated with Alexa Fluor 488 (Invitrogen, A-11034) (1:1000 in 0.5% FBS in PBS) for 1h at room temperature. The nuclei were stained with 0.3 mg/ml of **2-(4-Amidinophenyl)-6-indolecarbamidine dihydrochloride** (DAPI, Sigma, Israel). The cover glass slips were stuck to microscope slides using MOVIOL solution and observed under a Fluorescent Microscope with the appropriate filters for each fluorescent probe (Axio Observer Z1, Zeiss).
